# 4-(4-Methoxy­phen­yl)-3-[2-(2-methoxy­phen­yl)eth­yl]-1*H*-1,2,4-triazol-5(4*H*)-one

**DOI:** 10.1107/S1600536809002815

**Published:** 2009-01-31

**Authors:** Muhammad Hanif, Ghulam Qadeer, Nasim Hasan Rama, Javeed Akhtar, Madeleine Helliwell

**Affiliations:** a Department of Chemistry, Quaid-i-Azam University, Islamabad 45320, Pakistan; b The Manchester Materials Science Centre and Department of Chemistry, University of Manchester, Oxford Road, Manchester M13 9PL, England

## Abstract

The title compound, C_18_H_19_N_3_O_3_, is a biologically active triazole derivative. The five-membered ring is oriented with respect to the six-membered rings at dihedral angles of 51.59 (4) and 61.37 (4)°. The crystal structure is stabilized by inter­molecular N—H⋯O hydrogen-bond inter­actions between centrosymmetrically related mol­ecules [the dihedral angle between the benzene rings is 47.44 (5)°].

## Related literature

For the biological activities of triazole derivatives, see: Demirbas *et al.* (2002 ▶); Holla *et al.* (1998 ▶); Omar *et al.* (1986 ▶); Paulvannan *et al.* (2000 ▶); Turan-Zitouni *et al.* (1999 ▶); Kritsanida *et al.* (2002 ▶). For related structures, see: Öztürk *et al.* (2004*a* ▶,*b* ▶). For hydrogen-bond graph-set terminology, see: Bernstein *et al.* (1995 ▶); Etter (1990 ▶).
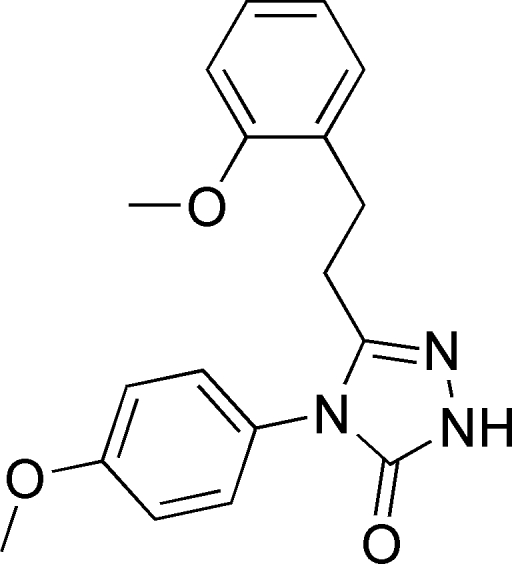


## Experimental

### 

#### Crystal data

C_18_H_19_N_3_O_3_
*M* *_r_* = 325.36
Monoclinic, 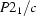

*a* = 12.5396 (19) Å
*b* = 9.1840 (14) Å
*c* = 14.041 (2) Å
β = 96.613 (3)°
*V* = 1606.3 (4) Å^3^
*Z* = 4
Mo *K*α radiation
μ = 0.09 mm^−1^
*T* = 100 (2) K
0.50 × 0.50 × 0.50 mm

#### Data collection

Bruker SMART CCD area-detector diffractometer
Absorption correction: none
8654 measured reflections
3278 independent reflections
2631 reflections with *I* > 2σ(*I*)
*R*_int_ = 0.031

#### Refinement

*R*[*F* ^2^ > 2σ(*F* ^2^)] = 0.032
*wR*(*F* ^2^) = 0.083
*S* = 1.01
3278 reflections
223 parameters
H atoms treated by a mixture of independent and constrained refinement
Δρ_max_ = 0.23 e Å^−3^
Δρ_min_ = −0.17 e Å^−3^

### 

Data collection: *SMART* (Bruker, 2001 ▶); cell refinement: *SAINT* (Bruker, 2002 ▶); data reduction: *SAINT*; program(s) used to solve structure: *SHELXS97* (Sheldrick, 2008 ▶); program(s) used to refine structure: *SHELXL97* (Sheldrick, 2008 ▶); molecular graphics: *SHELXTL* (Sheldrick, 2008 ▶); software used to prepare material for publication: *SHELXTL*.

## Supplementary Material

Crystal structure: contains datablocks global, I. DOI: 10.1107/S1600536809002815/rz2290sup1.cif

Structure factors: contains datablocks I. DOI: 10.1107/S1600536809002815/rz2290Isup2.hkl

Additional supplementary materials:  crystallographic information; 3D view; checkCIF report

## Figures and Tables

**Table 1:** Hydrogen-bond geometry (Å, °)

*D*—H⋯*A*	*D*—H	H⋯*A*	*D*⋯*A*	*D*—H⋯*A*
N3—H3N⋯O1^i^	0.913 (13)	1.870 (13)	2.7787 (12)	172.7 (12)

Symmetry code: (i) 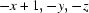.

**Fig. 1.**
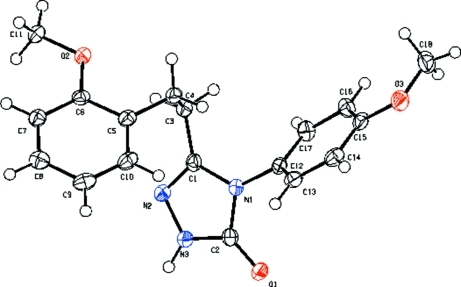
The molecular structure of the title compound with 50% probability displacement ellipsoids (arbitrary spheres for H atoms).

**Fig. 2.**
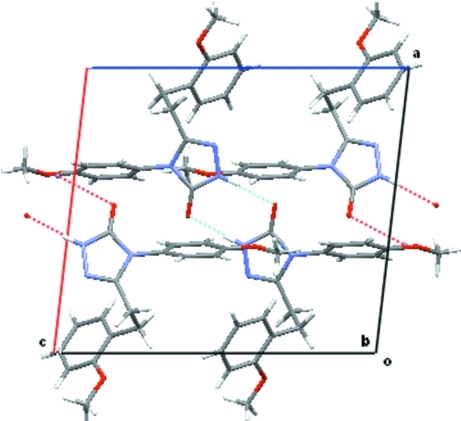
Crystal packing of the title compound viewed along the b axis. Hydrogen bonds are shown as dotted lines.

## supplementary crystallographic information

### Comment

Substituted triazole derivatives display significant biological activity including antimicrobial (Holla *et al.*, 1998), analgesic (Turan-Zitouni *et al.*, 1999), antitumor (Demirbas *et al.*, 2002), antihypertensive (Paulvannan *et al.*, 2000) and antiviral activities (Kritsanida *et al.*, 2002). The biological activity is closely related to the structure, possibly being due to the presence of the —N—C═S unit (Omar *et al.*, 1986). We are interested in the synthesis and biological activity of aryloxyacetyl hydrazide derivatives and report here the synthesis and crystal structure of the title compound (Fig. 1).

In the crystal structure of the title molecule, the bond lengths and angles are within normal range and comparable with those observed in related structures (Öztürk *et al.*, 2004*a*,*b*). In the triazole ring, the N3═C11 (1.2970 (14) Å) bond shows double bond character. The rings A (N1—N3/ C1/ C2), B (C5—C10) and C (C12—C17) are planar and the dihedral angles between them are A/B = 78.11 (3)°, A/C = 56.04 (3)° and B/C = 33.23 (3)°. In the crystal structure (Fig. 2), centrosymmetrically related molecules are linked by intermolecular N—H···O hydrogen bonds (Table 1) generating a ring of graph-set *R*^2^_2_(8) (Etter, 1990; Bernstein *et al.*,  1995).

### Experimental

The synthesis of the title compound was carried out by refluxing a solution of 4-(4-methoxyphenyl)-1-(3-(2-methoxyphenyl)propanoyl)semicarbazide (3.43 g, 10 mmol) in 2*M* NaOH for 5 h. Single crystals suitable for X-ray measurements were obtained on slow evaporation of an aqeous ethanol solution at room temperature (yield: 84%; m.p. 431–432 K).

### Refinement

H atoms bonded to C atoms were included in calculated positions and refined using the riding model approximation, with C—H = 0.95-0.99 Å and with *U*_iso_(H) = 1.2 *U*_eq_(C) or  1.5 *U*_eq_(C) for methyl H atoms. Atom H3N was located in a difference Fourier map and refined isotropically.

### Crystal data

**Table:**

C_18_H_19_N_3_O_3_	*F*(000) = 688
*M**_r_* = 325.36	*D*_x_ = 1.345 Mg m^−^^3^
Monoclinic, *P*2_1_/*c*	Melting point: 431(1) K
Hall symbol: -P 2ybc	Mo *K*α radiation, λ = 0.71073 Å
*a* = 12.5396 (19) Å	Cell parameters from 975 reflections
*b* = 9.1840 (14) Å	θ = 2.7–26.3°
*c* = 14.041 (2) Å	µ = 0.09 mm^−^^1^
β = 96.613 (3)°	*T* = 100 K
*V* = 1606.3 (4) Å^3^	Irregular, colourless
*Z* = 4	0.50 × 0.50 × 0.50 mm

### Data collection

**Table:**

Bruker SMART CCD area-detector diffractometer	2631 reflections with *I* > 2σ(*I*)
Radiation source: fine-focus sealed tube	*R*_int_ = 0.031
graphite	θ_max_ = 26.4°, θ_min_ = 2.7°
φ and ω scans	*h* = −15→14
8654 measured reflections	*k* = −7→11
3278 independent reflections	*l* = −17→17

### Refinement

**Table:**

Refinement on *F*^2^	Primary atom site location: structure-invariant direct methods
Least-squares matrix: full	Secondary atom site location: difference Fourier map
*R*[*F*^2^ > 2σ(*F*^2^)] = 0.032	Hydrogen site location: inferred from neighbouring sites
*wR*(*F*^2^) = 0.083	H atoms treated by a mixture of independent and constrained refinement
*S* = 1.01	*w* = 1/[σ^2^(*F*_o_^2^) + (0.0473*P*)^2^] where *P* = (*F*_o_^2^ + 2*F*_c_^2^)/3
3278 reflections	(Δ/σ)_max_ = 0.001
223 parameters	Δρ_max_ = 0.23 e Å^−^^3^
0 restraints	Δρ_min_ = −0.17 e Å^−^^3^

### Special details

**Table:**

Geometry. All e.s.d.'s (except the e.s.d. in the dihedral angle between two l.s. planes) are estimated using the full covariance matrix. The cell e.s.d.'s are taken into account individually in the estimation of e.s.d.'s in distances, angles and torsion angles; correlations between e.s.d.'s in cell parameters are only used when they are defined by crystal symmetry. An approximate (isotropic) treatment of cell e.s.d.'s is used for estimating e.s.d.'s involving l.s. planes.
Refinement. Refinement of *F*^2^ against ALL reflections. The weighted *R*-factor *wR* and goodness of fit *S* are based on *F*^2^, conventional *R*-factors *R* are based on *F*, with *F* set to zero for negative *F*^2^. The threshold expression of *F*^2^ > σ(*F*^2^) is used only for calculating *R*-factors(gt) *etc*. and is not relevant to the choice of reflections for refinement. *R*-factors based on *F*^2^ are statistically about twice as large as those based on *F*, and *R*- factors based on ALL data will be even larger.

### Fractional atomic coordinates and isotropic or equivalent isotropic displacement parameters (Å^2^)

**Table:**

	*x*	*y*	*z*	*U*_iso_*/*U*_eq_	
O1	0.48159 (6)	0.03096 (9)	0.13337 (5)	0.0255 (2)	
O2	1.08680 (6)	−0.01642 (9)	0.16392 (5)	0.0249 (2)	
O3	0.62644 (6)	0.09499 (10)	0.58898 (5)	0.0306 (2)	
N1	0.65632 (7)	−0.02461 (11)	0.20442 (6)	0.0205 (2)	
N2	0.72808 (7)	−0.09381 (11)	0.07470 (6)	0.0227 (2)	
N3	0.62158 (7)	−0.05346 (11)	0.05209 (7)	0.0224 (2)	
H3N	0.5916 (10)	−0.0520 (14)	−0.0104 (9)	0.029 (3)*	
C1	0.74701 (9)	−0.07600 (12)	0.16673 (8)	0.0210 (3)	
C2	0.57538 (9)	−0.01011 (13)	0.12919 (8)	0.0209 (3)	
C3	0.85367 (9)	−0.09907 (14)	0.22308 (8)	0.0249 (3)	
H3A	0.9001	−0.1548	0.1837	0.030*	
H3B	0.8442	−0.1579	0.2806	0.030*	
C4	0.90982 (9)	0.04491 (14)	0.25474 (8)	0.0260 (3)	
H4A	0.8621	0.1023	0.2919	0.031*	
H4B	0.9762	0.0230	0.2976	0.031*	
C5	0.93810 (8)	0.13522 (13)	0.17195 (7)	0.0224 (3)	
C6	1.02928 (9)	0.10111 (13)	0.12649 (7)	0.0209 (3)	
C7	1.05663 (9)	0.18328 (13)	0.05035 (8)	0.0226 (3)	
H7	1.1184	0.1591	0.0202	0.027*	
C8	0.99314 (9)	0.30150 (13)	0.01822 (9)	0.0262 (3)	
H8	1.0122	0.3589	−0.0335	0.031*	
C9	0.90262 (9)	0.33607 (14)	0.06091 (9)	0.0284 (3)	
H9	0.8588	0.4160	0.0381	0.034*	
C10	0.87625 (9)	0.25331 (13)	0.13719 (8)	0.0264 (3)	
H10	0.8141	0.2780	0.1666	0.032*	
C11	1.17827 (9)	−0.05778 (13)	0.11798 (8)	0.0252 (3)	
H11A	1.1559	−0.0769	0.0499	0.038*	
H11B	1.2104	−0.1460	0.1484	0.038*	
H11C	1.2312	0.0212	0.1242	0.038*	
C12	0.64536 (8)	0.00320 (13)	0.30338 (8)	0.0202 (3)	
C13	0.62172 (8)	0.14267 (13)	0.33266 (8)	0.0232 (3)	
H13	0.6103	0.2192	0.2871	0.028*	
C14	0.61483 (9)	0.16956 (14)	0.42864 (8)	0.0260 (3)	
H14	0.5978	0.2646	0.4490	0.031*	
C15	0.63274 (8)	0.05796 (14)	0.49543 (8)	0.0236 (3)	
C16	0.65578 (9)	−0.08137 (14)	0.46578 (8)	0.0256 (3)	
H16	0.6684	−0.1578	0.5113	0.031*	
C17	0.66031 (9)	−0.10809 (13)	0.36918 (8)	0.0251 (3)	
H17	0.6738	−0.2040	0.3482	0.030*	
C18	0.64879 (10)	−0.01663 (16)	0.65935 (8)	0.0344 (3)	
H18A	0.6017	−0.1002	0.6428	0.052*	
H18B	0.6362	0.0209	0.7224	0.052*	
H18C	0.7239	−0.0471	0.6610	0.052*	

### Atomic displacement parameters (Å^2^)

**Table:**

	*U*^11^	*U*^22^	*U*^33^	*U*^12^	*U*^13^	*U*^23^
O1	0.0190 (4)	0.0342 (5)	0.0238 (4)	0.0022 (3)	0.0042 (3)	−0.0020 (4)
O2	0.0212 (4)	0.0306 (5)	0.0238 (4)	0.0073 (3)	0.0060 (3)	0.0039 (4)
O3	0.0274 (5)	0.0434 (6)	0.0213 (4)	−0.0008 (4)	0.0043 (3)	−0.0082 (4)
N1	0.0190 (5)	0.0238 (5)	0.0192 (5)	0.0008 (4)	0.0046 (4)	0.0017 (4)
N2	0.0199 (5)	0.0247 (5)	0.0238 (5)	0.0026 (4)	0.0041 (4)	0.0012 (4)
N3	0.0199 (5)	0.0281 (6)	0.0193 (5)	0.0018 (4)	0.0029 (4)	−0.0001 (4)
C1	0.0223 (6)	0.0192 (6)	0.0226 (6)	0.0008 (5)	0.0068 (5)	0.0027 (5)
C2	0.0215 (6)	0.0196 (6)	0.0219 (6)	−0.0022 (5)	0.0040 (5)	0.0015 (5)
C3	0.0223 (6)	0.0308 (7)	0.0223 (6)	0.0062 (5)	0.0058 (5)	0.0052 (5)
C4	0.0190 (6)	0.0409 (8)	0.0181 (5)	0.0052 (5)	0.0018 (4)	−0.0032 (5)
C5	0.0183 (6)	0.0277 (6)	0.0204 (5)	−0.0006 (5)	−0.0008 (4)	−0.0076 (5)
C6	0.0187 (6)	0.0232 (6)	0.0199 (5)	0.0011 (5)	−0.0018 (4)	−0.0034 (5)
C7	0.0199 (6)	0.0254 (7)	0.0222 (6)	−0.0017 (5)	0.0017 (4)	−0.0043 (5)
C8	0.0268 (6)	0.0235 (6)	0.0274 (6)	−0.0042 (5)	−0.0010 (5)	0.0002 (5)
C9	0.0244 (6)	0.0227 (6)	0.0365 (7)	0.0036 (5)	−0.0031 (5)	−0.0026 (6)
C10	0.0200 (6)	0.0285 (7)	0.0305 (6)	0.0025 (5)	0.0013 (5)	−0.0092 (6)
C11	0.0203 (6)	0.0290 (7)	0.0269 (6)	0.0054 (5)	0.0056 (5)	0.0001 (5)
C12	0.0164 (5)	0.0249 (6)	0.0200 (6)	−0.0015 (5)	0.0043 (4)	−0.0010 (5)
C13	0.0185 (6)	0.0230 (6)	0.0280 (6)	0.0003 (5)	0.0018 (5)	0.0025 (5)
C14	0.0216 (6)	0.0254 (6)	0.0307 (6)	0.0020 (5)	0.0024 (5)	−0.0065 (5)
C15	0.0153 (6)	0.0336 (7)	0.0222 (6)	−0.0039 (5)	0.0037 (4)	−0.0064 (5)
C16	0.0288 (6)	0.0265 (7)	0.0222 (6)	−0.0030 (5)	0.0052 (5)	0.0036 (5)
C17	0.0294 (6)	0.0219 (6)	0.0252 (6)	−0.0011 (5)	0.0074 (5)	−0.0010 (5)
C18	0.0325 (7)	0.0503 (9)	0.0207 (6)	−0.0115 (6)	0.0048 (5)	−0.0026 (6)

### Geometric parameters (Å, °)

**Table:**

O1—C2	1.2429 (13)	C7—H7	0.9500
O2—C6	1.3690 (14)	C8—C9	1.3802 (16)
O2—C11	1.4305 (13)	C8—H8	0.9500
O3—C15	1.3679 (13)	C9—C10	1.3840 (17)
O3—C18	1.4290 (16)	C9—H9	0.9500
N1—C2	1.3844 (14)	C10—H10	0.9500
N1—C1	1.3909 (14)	C11—H11A	0.9800
N1—C12	1.4348 (14)	C11—H11B	0.9800
N2—C1	1.2970 (14)	C11—H11C	0.9800
N2—N3	1.3870 (13)	C12—C17	1.3761 (16)
N3—C2	1.3455 (14)	C12—C13	1.3877 (17)
N3—H3N	0.913 (13)	C13—C14	1.3825 (16)
C1—C3	1.4886 (15)	C13—H13	0.9500
C3—C4	1.5394 (17)	C14—C15	1.3899 (17)
C3—H3A	0.9900	C14—H14	0.9500
C3—H3B	0.9900	C15—C16	1.3862 (17)
C4—C5	1.5033 (16)	C16—C17	1.3859 (16)
C4—H4A	0.9900	C16—H16	0.9500
C4—H4B	0.9900	C17—H17	0.9500
C5—C10	1.3888 (16)	C18—H18A	0.9800
C5—C6	1.4074 (15)	C18—H18B	0.9800
C6—C7	1.3836 (16)	C18—H18C	0.9800
C7—C8	1.3906 (17)		
			
C6—O2—C11	116.79 (8)	C7—C8—H8	119.8
C15—O3—C18	117.21 (10)	C8—C9—C10	119.44 (11)
C2—N1—C1	107.58 (9)	C8—C9—H9	120.3
C2—N1—C12	125.32 (9)	C10—C9—H9	120.3
C1—N1—C12	127.05 (9)	C9—C10—C5	121.82 (11)
C1—N2—N3	104.79 (9)	C9—C10—H10	119.1
C2—N3—N2	112.79 (9)	C5—C10—H10	119.1
C2—N3—H3N	126.9 (8)	O2—C11—H11A	109.5
N2—N3—H3N	120.1 (8)	O2—C11—H11B	109.5
N2—C1—N1	110.97 (10)	H11A—C11—H11B	109.5
N2—C1—C3	124.18 (10)	O2—C11—H11C	109.5
N1—C1—C3	124.75 (10)	H11A—C11—H11C	109.5
O1—C2—N3	128.70 (10)	H11B—C11—H11C	109.5
O1—C2—N1	127.42 (10)	C17—C12—C13	120.26 (11)
N3—C2—N1	103.87 (9)	C17—C12—N1	119.81 (10)
C1—C3—C4	112.60 (10)	C13—C12—N1	119.92 (10)
C1—C3—H3A	109.1	C14—C13—C12	119.55 (11)
C4—C3—H3A	109.1	C14—C13—H13	120.2
C1—C3—H3B	109.1	C12—C13—H13	120.2
C4—C3—H3B	109.1	C13—C14—C15	120.17 (11)
H3A—C3—H3B	107.8	C13—C14—H14	119.9
C5—C4—C3	113.04 (9)	C15—C14—H14	119.9
C5—C4—H4A	109.0	O3—C15—C16	123.70 (11)
C3—C4—H4A	109.0	O3—C15—C14	116.22 (11)
C5—C4—H4B	109.0	C16—C15—C14	120.08 (11)
C3—C4—H4B	109.0	C17—C16—C15	119.39 (11)
H4A—C4—H4B	107.8	C17—C16—H16	120.3
C10—C5—C6	117.67 (11)	C15—C16—H16	120.3
C10—C5—C4	122.05 (10)	C12—C17—C16	120.52 (11)
C6—C5—C4	120.28 (10)	C12—C17—H17	119.7
O2—C6—C7	124.06 (10)	C16—C17—H17	119.7
O2—C6—C5	114.90 (10)	O3—C18—H18A	109.5
C7—C6—C5	121.04 (11)	O3—C18—H18B	109.5
C6—C7—C8	119.53 (11)	H18A—C18—H18B	109.5
C6—C7—H7	120.2	O3—C18—H18C	109.5
C8—C7—H7	120.2	H18A—C18—H18C	109.5
C9—C8—C7	120.49 (12)	H18B—C18—H18C	109.5
C9—C8—H8	119.8		
			
C1—N2—N3—C2	0.48 (13)	O2—C6—C7—C8	−179.48 (10)
N3—N2—C1—N1	−0.33 (12)	C5—C6—C7—C8	0.05 (17)
N3—N2—C1—C3	−176.87 (11)	C6—C7—C8—C9	−0.81 (17)
C2—N1—C1—N2	0.10 (13)	C7—C8—C9—C10	0.98 (17)
C12—N1—C1—N2	177.68 (10)	C8—C9—C10—C5	−0.40 (18)
C2—N1—C1—C3	176.61 (11)	C6—C5—C10—C9	−0.34 (17)
C12—N1—C1—C3	−5.81 (18)	C4—C5—C10—C9	−179.98 (11)
N2—N3—C2—O1	−179.17 (11)	C2—N1—C12—C17	117.56 (13)
N2—N3—C2—N1	−0.41 (12)	C1—N1—C12—C17	−59.61 (15)
C1—N1—C2—O1	178.97 (11)	C2—N1—C12—C13	−63.64 (15)
C12—N1—C2—O1	1.34 (19)	C1—N1—C12—C13	119.18 (13)
C1—N1—C2—N3	0.19 (12)	C17—C12—C13—C14	0.94 (17)
C12—N1—C2—N3	−177.45 (10)	N1—C12—C13—C14	−177.85 (10)
N2—C1—C3—C4	105.59 (13)	C12—C13—C14—C15	0.76 (17)
N1—C1—C3—C4	−70.47 (14)	C18—O3—C15—C16	2.24 (16)
C1—C3—C4—C5	−64.75 (13)	C18—O3—C15—C14	−177.56 (10)
C3—C4—C5—C10	100.59 (13)	C13—C14—C15—O3	178.71 (10)
C3—C4—C5—C6	−79.05 (13)	C13—C14—C15—C16	−1.10 (17)
C11—O2—C6—C7	−2.34 (15)	O3—C15—C16—C17	179.96 (10)
C11—O2—C6—C5	178.10 (9)	C14—C15—C16—C17	−0.26 (17)
C10—C5—C6—O2	−179.92 (9)	C13—C12—C17—C16	−2.32 (17)
C4—C5—C6—O2	−0.27 (15)	N1—C12—C17—C16	176.47 (10)
C10—C5—C6—C7	0.51 (16)	C15—C16—C17—C12	1.96 (17)
C4—C5—C6—C7	−179.84 (10)		

### Hydrogen-bond geometry (Å, °)

**Table:**

*D*—H···*A*	*D*—H	H···*A*	*D*···*A*	*D*—H···*A*
N3—H3N···O1^i^	0.913 (13)	1.870 (13)	2.7787 (12)	172.7 (12)

Symmetry codes: (i) −*x*+1, −*y*, −*z*.

## References

[bb1] Bernstein, J., Davis, R. E., Shimoni, L. & Chang, N.-L. (1995). *Angew. Chem. Int. Ed. Engl.* **34**, 1555-1573.

[bb2] Bruker (2001). *SMART* Bruker AXS Inc., Madison, Wisconsin, USA.

[bb3] Bruker (2002). *SAINT* Bruker AXS Inc., Madison, Wisconsin, USA.

[bb4] Demirbas, N., Ugurluoglu, R. & Demirbas, A. (2002). *Bioorg. Med. Chem.* **10**, 3717–3723. 10.1016/s0968-0896(02)00420-0 12413828

[bb5] Etter, M. C. (1990). *Acc. Chem. Res.* **23**, 120–126.

[bb6] Holla, B. S., Gonsalves, R. & Shenoy, S. (1998). * Farmaco*, **53**, 574–578. 10.1016/s0014-827x(98)00068-8 10081820

[bb7] Kritsanida, M., Mouroutsou, A., Marakos, P., Pouli, N., Papakonstantinou- Garoufalias, S., Pannecouque, C., Witvrouw, M. & Clercq, E. D. (2002). *Farmaco*, **57**, 253–257. 10.1016/s0014-827x(01)01189-2 11989804

[bb8] Omar, A., Mohsen, M. E. & Wafa, O. A. (1986). *Heterocycl. Chem.* **23**, 1339–1341.

[bb9] Öztürk, S., Akkurt, M., Cansız, A., Koparır, M., Şekerci, M. & Heinemann, F. W. (2004*a*). *Acta Cryst.* E**60**, o425–o427.

[bb10] Öztürk, S., Akkurt, M., Cansız, A., Koparır, M., Şekerci, M. & Heinemann, F. W. (2004*b*). *Acta Cryst.* E**60**, o642–o644.

[bb11] Paulvannan, K., Chen, T. & Hale, R. (2000). *Tetrahedron*, **56**, 8071–8076.

[bb12] Sheldrick, G. M. (2008). *Acta Cryst.* A**64**, 112–122. 10.1107/S0108767307043930 18156677

[bb13] Turan-Zitouni, G., Kaplancikli, Z. A., Erol, K. & Kilic, F. S. (1999). *Farmaco*, **54**, 218–223. 10.1016/s0014-827x(99)00016-6 10384714

